# The oncolytic peptide LTX-315 kills cancer cells through Bax/Bak-regulated mitochondrial membrane permeabilization

**DOI:** 10.18632/oncotarget.5613

**Published:** 2015-09-10

**Authors:** Heng Zhou, Sabrina Forveille, Allan Sauvat, Valentina Sica, Valentina Izzo, Sylvère Durand, Kevin Müller, Peng Liu, Laurence Zitvogel, Øystein Rekdal, Oliver Kepp, Guido Kroemer

**Affiliations:** ^1^ Metabolomics and Cell Biology Platforms, Gustave Roussy Comprehensive Cancer Institute, Villejuif, France; ^2^ Equipe 11 Labellisée Ligue Contre le Cancer, Centre de Recherche des Cordeliers, INSERM U 1138, Paris, France; ^3^ Université Paris Descartes, Sorbonne Paris Cité, Paris, France; ^4^ Université Pierre et Marie Curie, Paris, France; ^5^ University of Paris Sud XI, Kremlin Bicêtre, France; ^6^ Department of Immuno-Oncology, Institut de Cancérologie Gustave Roussy Cancer Campus, Villejuif, France; ^7^ Institut National de la Santé et de la Recherche Medicale (INSERM), U1015, Villejuif, France; ^8^ Center of Clinical Investigations in Biotherapies of Cancer (CICBT) 507, Villejuif, France; ^9^ Lytix Biopharma, Oslo, Norway; ^10^ University of Tromsø, Institute of Medical Biology, Tromsø, Norway; ^11^ Pôle de Biologie, Hôpital Européen Georges Pompidou, AP-HP, Paris, France; ^12^ Karolinska Institute, Department of Women’s and Children’s Health, Karolinska University Hospital, Stockholm, Sweden

**Keywords:** LTX-315, necrosis, mitochondrial membrane permeabilization, cancer, mitophagy

## Abstract

LTX-315 has been developed as an amphipathic cationic peptide that kills cancer cells. Here, we investigated the putative involvement of mitochondria in the cytotoxic action of LTX-315. Subcellular fractionation of LTX-315-treated cells, followed by mass spectrometric quantification, revealed that the agent was enriched in mitochondria. LTX-315 caused an immediate arrest of mitochondrial respiration without any major uncoupling effect. Accordingly, LTX-315 disrupted the mitochondrial network, dissipated the mitochondrial inner transmembrane potential, and caused the release of mitochondrial intermembrane proteins into the cytosol. LTX-315 was relatively inefficient in stimulating mitophagy. Cells lacking the two pro-apoptotic multidomain proteins from the BCL-2 family, BAX and BAK, were less susceptible to LTX-315-mediated killing. Moreover, cells engineered to lose their mitochondria (by transfection with Parkin combined with treatment with a protonophore causing mitophagy) were relatively resistant against LTX-315, underscoring the importance of this organelle for LTX-315-mediated cytotoxicity. Altogether, these results support the notion that LTX-315 kills cancer cells by virtue of its capacity to permeabilize mitochondrial membranes.

## INTRODUCTION

Cancer cells heavily rely on mitochondrial metabolism due to their particular growth characteristics [1-4], implying that targeting mitochondria may constitute a relevant strategy for reducing proliferation or killing tumor cells [5-7]. Accordingly, multiple anticancer agents have been developed with the scope of targeting mitochondria.

Among such mitochondriotropic agents, those endowed with the capacity of permeabilizing mitochondrial membranes are particularly interesting [5, 6, 8]. Such agents can stimulate the intrinsic apoptotic pathway (which involves an obligate step of mitochondrial outer membrane permeabilization, MOMP) in a direct fashion [9]. Hence, instead of indirectly eliciting MOMP downstream of organellar stress pathways, usually the DNA damage response (which is frequently deactivated in cancer cells, causing drug resistance), mitochondrion-targeted anticancer agents can directly stimulate MOMP thereby kicking off the apoptotic cascade [9, 10]. Hence direct MOMP inducers can ignite cell death pathways downstream of the usual roadblocks (such as inactivation of the p53 pathway) that mediate resistance to many anticancer agents.

Cationic amphiphilic agents can induce mitochondrial permeabilization by virtue of their capacity to selectively enrich at the level of the inner mitochondrial membrane, as a result of their distribution following electrochemical gradients. Such agents incorporate into mitochondria, first driven by the plasma membrane potential and then again by the local transmembrane potential (Δψ_m_), following the Nernst equation [11-16]. Cationic amphiphilic agents exist naturally as antimicrobial peptides with broad-spectrum antibiotic activity, as exemplified by bovine lactoferricin. Since lactoferricin also mediates anticancer effects [17-19], efforts have been undertaken to generate lactoferricin analogues with improved oncolytic activity, giving rise to the design of LTX-315 a modified synthetic peptide that is currently undergoing clinical trials for the treatment of advanced cancer. LTX-315 has the particularity to induce the regression of B16 melanomas *in vivo* upon its local injection into the tumor [20]. This effect is accompanied by the infiltration of the tumor by T lymphocytes and the elicitation of an anticancer immune response.

Here we addressed the question as to whether LTX-315 truly targets the mitochondrial compartment for cell death induction or whether this agent may act through additional (off-target) effects. The results of our work reveal multiple pieces of evidence indicating that LTX-315 acts on-target, via the permeabilization of mitochondria, thereby killing cancer cells.

## RESULTS AND DISCUSSION

### Mitochondrial enrichment and effects of LTX-315

**Figure 1:**
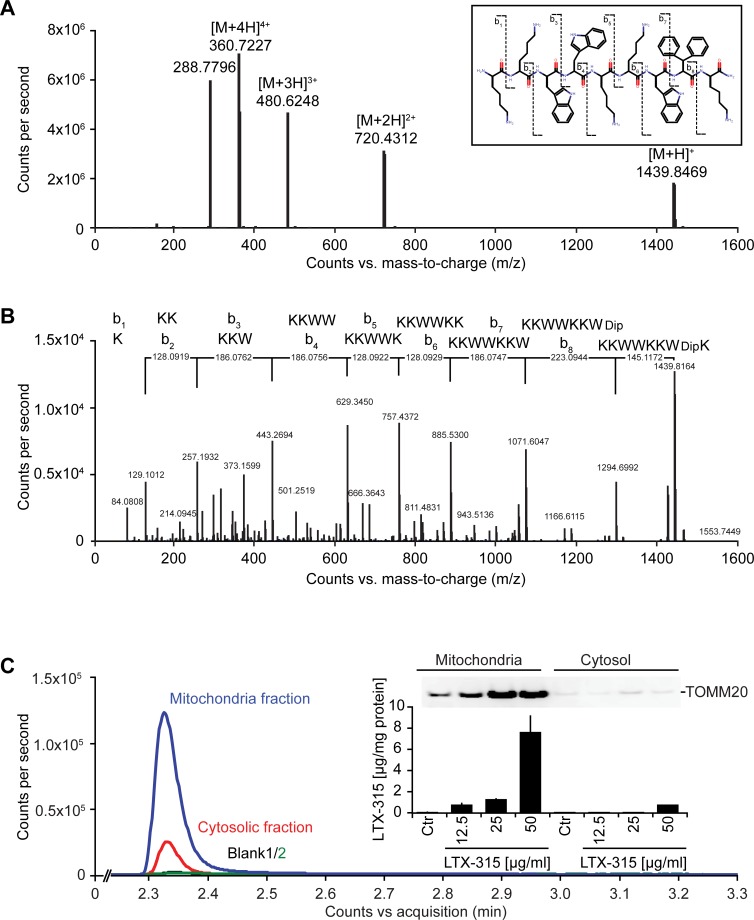
Mass spectrometric detection of LTX-315 enriched in the mitochondrial fraction **A.** Full scan mass spectrum of LTX-315 (C_78_H_106_N_18_O_9_) revealed the scattered structure of the peptide, revealing its 4 protonation levels, that yield in signals used for quantification. **B.** Selection and fragmentation of the [M+H]^+^. The peptide sequence is analyzed by ESI-HRMS following a standardized fragmentation pattern. **C.** Subcellular fractionation yielded in cytoplasmic and mitochondrial fractions that were tested for purity by immunobloting using mitochondria-specific TOMM20 antibody. Each fraction was analyzed and yielded in chromatographic peaks of the LTX-315 in the mitochondria and cytosolic fractions with different amplitudes. Subsequently the concentration of LTX-315 peptide was evaluated by BSA protein quantification in each fraction.

**Figure 2:**
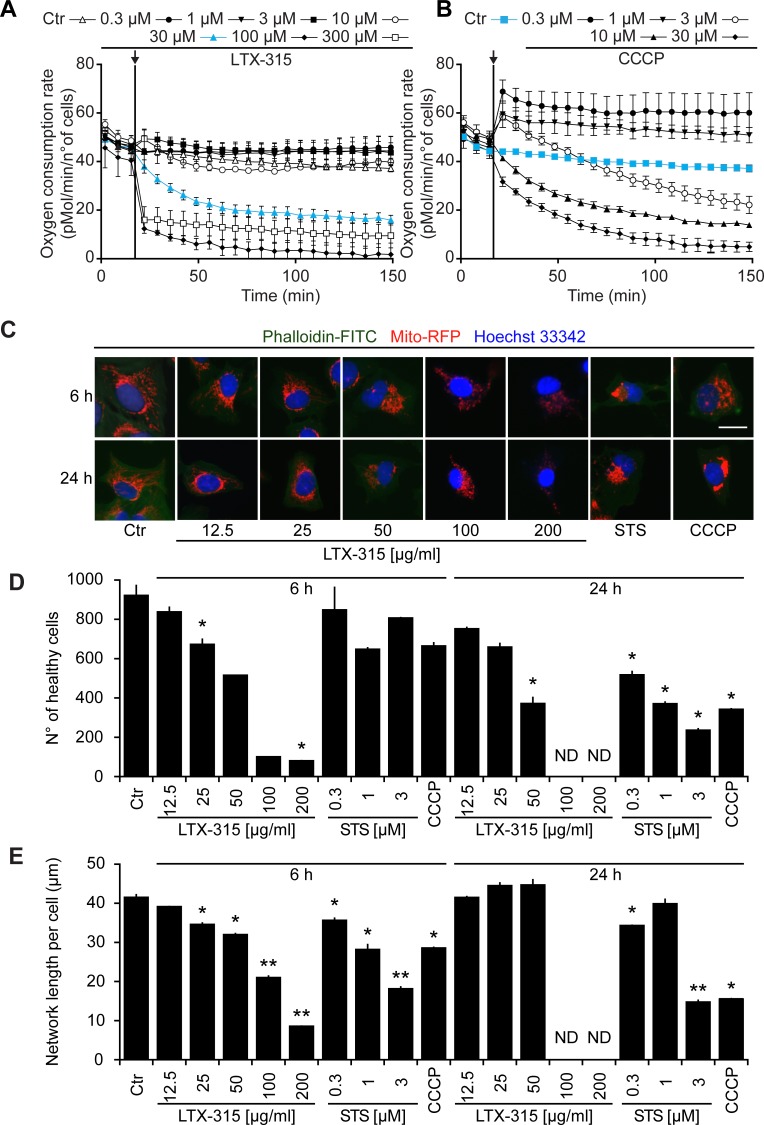
Functional and morphological disruption of mitochondria by LTX-315 **A.**, **B.** Effects of LTX-315 and CCCP on mitochondrial respiration. Cells were cultured in specialized XF-96-well plates, and the indicated concentrations of LTX-315 **A.** or CCCP **B.** were added, as pointed at by the arrows. Oxygen consumption was monitored continuously in a Seahorse apparatus. Results are means ± SD of hexaplicates. **C.**, **D.** Effects of LTX-315 on cellular viability and mitochondrial morphology. U2OS cells stably transfected with a mitochondrion-targeted RFP were cultured for 6 or 24 h with the indicated concentrations of LTX, STS (standard dose: 1 μM) or CCCP (standard dose: 10 μM), and then counterstained for the detection of the F-actin cytoskeleton with FITC-labelled phalloidin and the visualization of chromatin with Hoechst 33342. Representative images of cells with intact nuclear and cytoplasmic morphology are shown in **C.** Cells with a apparently normal morphology (intact nuclei + cytoplasm) were counted by automated microscopy **D.**, and the mean length of discernible mitochondrial networks per cells was determined by morphometric image analyses **E.** Results are means ± SD of triplicates. Asterisks indicate significant (unpaired Welch’s *t* test) changes with respect to untreated controls (Ctr). *p < 0.05; **p < 0.01; ***p < 0.001. Size bar equals 10 μm.

LTX-315 is a peptide derivative (insert in Figure 1A), that can be detected by mass spectrometry (Figure 1A), including after its collisional fragmentation giving rise to smaller masses (Figure 1B). In cells that were exposed to doses of LTX-315 that are non-toxic (12.5 to 25 μg/ml) or only kill a fraction of cells (50 μg/ml, see below), LTX-315 was clearly enriched in the mitochondrial as opposed to the cytosolic fraction (Figure 1C), supporting the notion that this amphipathic cationic peptide readily reaches its target organelle. Accordingly, LTX-315 caused a close-to-immediate cessation of mitochondrial respiration when added to cells at concentrations ranging from 30 μg/ml to 300 μg/ml (Figure 2A). This effect was even more abrupt than the one obtained with high doses (10-30 μM) of the protonophore carbonyl cyanide m-chlorophenyl hydrazine (CCCP) (Figure 2B). As compared to CCCP, which increased respiration at low doses (0.3 to 1 μM), low doses of LTX-315 (0.3 μg/ml to 10 μg/ml) failed to stimulate oxygen consumption (Figure 2A, 2B, Supplemental Figure 1), indicating that LTX-315 is devoid of any uncoupling effect. When added to U2OS osteosarcoma cells at variable concentrations (12.5 to 200 μg/ml) and intervals (6 to 24 h), LTX-315 was found to kill close-to all cells at doses ≥100 μg/ml and to mediate partial cytotoxic effects at 25 to 50 μg/ml, meaning that cells bearing a close-to-normal morphology (with Hoechst 33342-detectable chromatin and a phalloidin-FITC-reactive F-actin cytoskeleton) were still detectable (Figure 2B, 2C). In contrast, LTX-315 only mediated significant erythrocyte lysis at doses >200 μg/ml (Supplemental Figure 2), supporting the idea that direct detergent-like effects on the plasma membrane are unlikely to explain the cytotoxic action of LTX-315. In addition, LTX-315 disrupted the tubular mitochondrial network (labeled by stable transfection with a mitochondrion-located red fluorescent protein, RFP) in still intact cells, causing its fragmentation. This effect, which was measured by fluorescence microscopy and morphometric analysis, was particularly pronounced at short time points (Figure 2B, 2D), supporting the mitochondriotoxic action of LTX-315.

### Mitochondrial permeabilization by LTX-315

**Figure 3:**
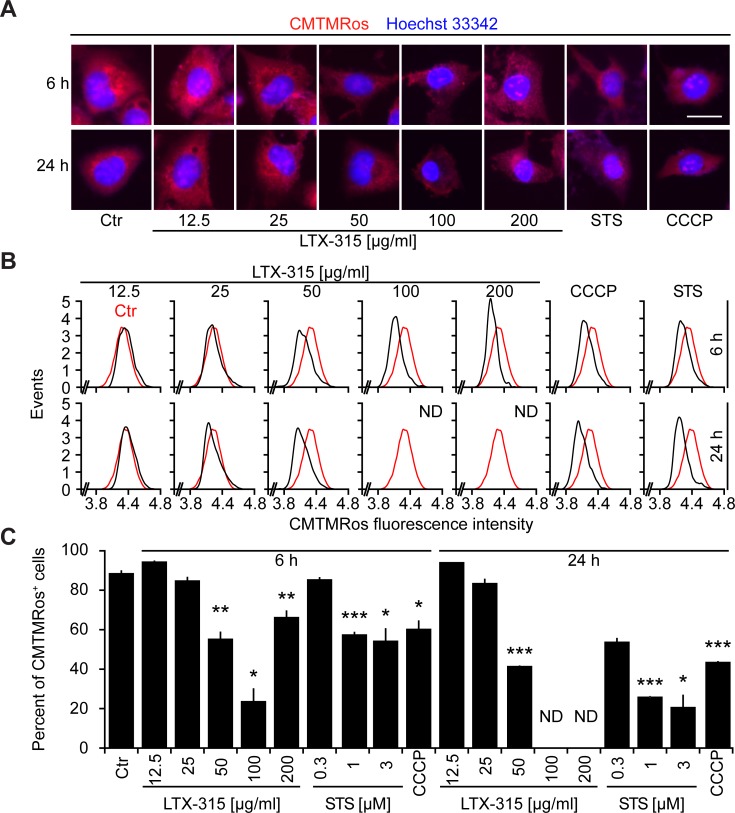
Dissipation of the mitochondrial transmembrane potential (Δψ_m_) by LTX-315 U2OS cells were cultured for 6 or 24 h with the indicated concentrations of LTX-315, STS (standard dose: 1 μM) or CCCP (standard dose: 10 μM) and then subjected to staining with the Δψ_m_-sensitive fluorochrome CMTMRos (which incorporates into the mitochondrial matrix driven by the electrochemical gradient) and counterstained with Hoechst 33342. Representative microphotographs are shown in **A.** The distribution of the CMTMRos staining intensity for morphologically normal cells is shown in **B.** Finally, quantitative results showing the frequency of cells with low CMTMRos incorporation (<4.4 in B) are given in **C.** Columns indicate means ± SD of triplicates. Asterisks indicate significant (unpaired Welch’s *t* test) changes with respect to untreated controls (Ctr). *p < 0.05; **p < 0.01; ***p < 0.001. Size bar equals 10 μm.

**Figure 4:**
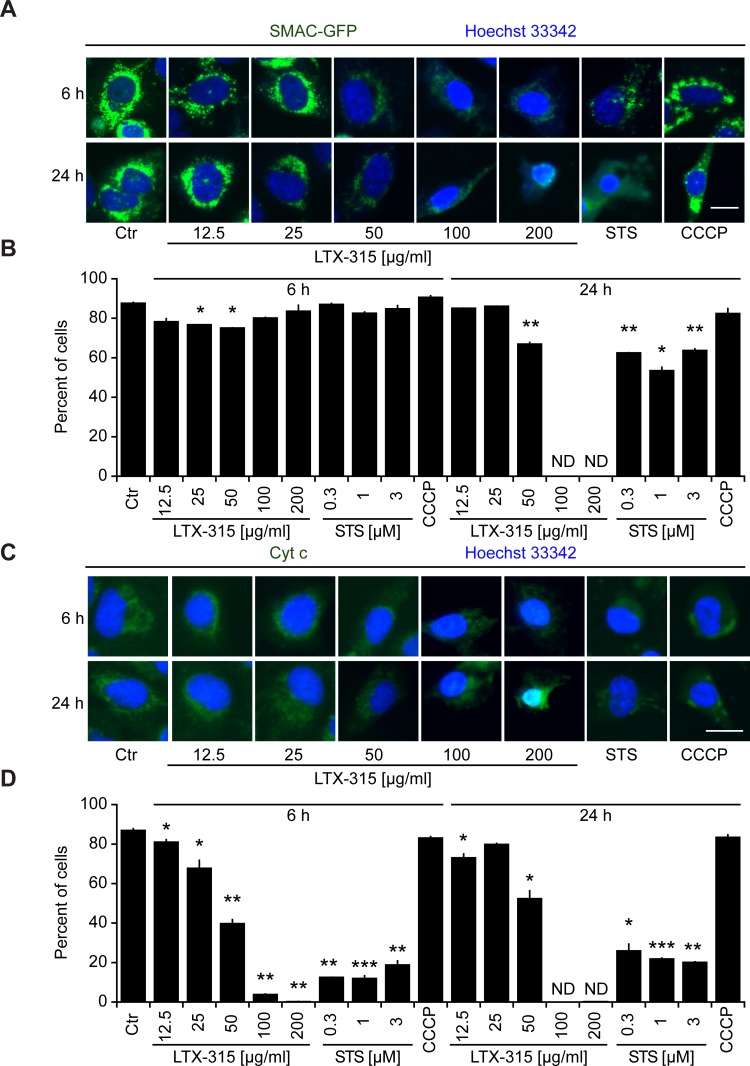
Mitochondrial outer membrane permeabilization induced by LTX-315 **A.**, **B.** Release of SMAC from mitochondria to the cytosol. U2OS cells stably transfected with SMAC-GFP fusion protein cultured for 6 or 24 h with the indicated concentrations of LTX-315, STS (standard dose: 1 μM) or CCCP (standard dose: 10 μM) and then counterstained with Hoechst 33342. **C.**, **D.** Release of cytochrome *c* from mitochondria. Untransfected U2OS cells were cultured as in A and B and subjected to immunofluorescence detection of cytochrome *c* and counterstaining with Hoechst 33342. Representative pictures of still intact cells are shown in **A.** and **C.** Quantitative results are shown in **B.** and **D.** Columns indicate means ± SD of triplicates. Asterisks indicate significant (unpaired Welch’s *t* test) changes with respect to untreated controls (Ctr). *p < 0.05; **p < 0.01; ***p < 0.001. Size bar equals 10 μm.

Cells exposed to doses of LTX-315 ranging from 50 to 200 μg/ml exhibited the dissipation of the inner mitochondrial transmembrane potential (Δψ_m_), as detectable with the Δψ_m_-sensitive dye chloromethyltetramethylrosamine (CMTMRos), a cationic lipophilic fluorochrome that incorporated into the mitochondrial matrix driven by the Nernst equation [21, 22]. This LTX-315 effect was comparable to the Δψ_m_ dissipation mediated by the protonophore CCCP or the pro-apoptotic pan-tyrosine kinase inhibitor staurosporine (STS) (Figure 3). Since Δψ_m_ dissipation is often associated with the permeabilization of the outer mitochondrial membrane [23, 24], we next investigated whether LTX-315 can liberate intermembrane proteins such as SMAC and cytochrome *c* from mitochondria. Indeed, LTX-315 caused the mitochondrial release of a SMAC-GFP fusion protein stably expressed by U2OS cells, meaning that SMAC-GFP lost its granular distribution and became detectable throughout the cytosol (Figure 4A, 4B). These results could be recapitulated for cytochrome *c*, which was detected by immunofluorescence staining of fixed and permeabilized cells. Again, doses of LTX-315 that left most of the cells intact (12.5-25 μg/ml) or only caused partial cell killing (50 μg/ml) induced a reduction of the mitochondrial (granular) abundance of cytochrome *c* that was detectable in cells that otherwise manifested a normal morphology (Figure 4C, 4D). With this respect, LTX-315 behaves similar to the pro-apoptotic agent staurosporine, which also induced tangible signs of outer mitochondrial membrane permeabilization.

**Figure 5:**
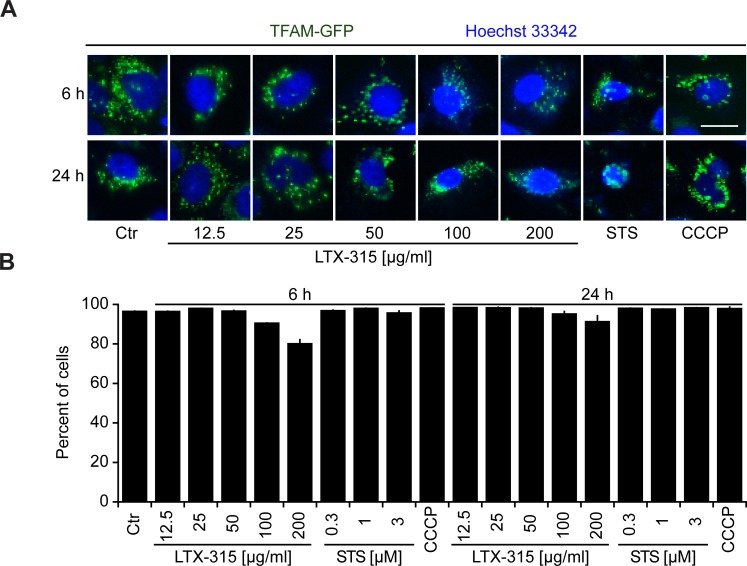
Retention of TFAM in mitochondria **A.**, **B.** Retention of TFAM in mitochondria. U2OS cells stably expressing a TFAM-GFP fusion protein were cultured for 6 or 24 h with the indicated concentrations of LTX-315, STS (standard dose: 1 μM) or CCCP (standard dose: 10 μM) and then counterstained with Hoechst 33342. Representative pictures of still intact cells are shown. **A.** Columns indicate means ± SD of triplicates. Asterisks indicate significant (unpaired Welch’s *t* test) changes with respect to untreated controls (Ctr). *p < 0.05; **p < 0.01; ***p < 0.001. Size bar equals 10 μm.

We also generated a U2OS clone stably expressing a TFAM (transcription factor A, mitochondrial)-GFP fusion protein. TFAM is a matrix protein interacting with mitochondrial DNA [25]. In contrast to SMAC-GFP or cytochrome *c*, the granular TFAM staining did not disappear completely (Figure 5A, 5B) indicating the persistence of remnants of the mitochondrial structure that retain TFAM.

### Impact of BCL-2 family proteins on LTX-315-induced cell killing

**Figure 6:**
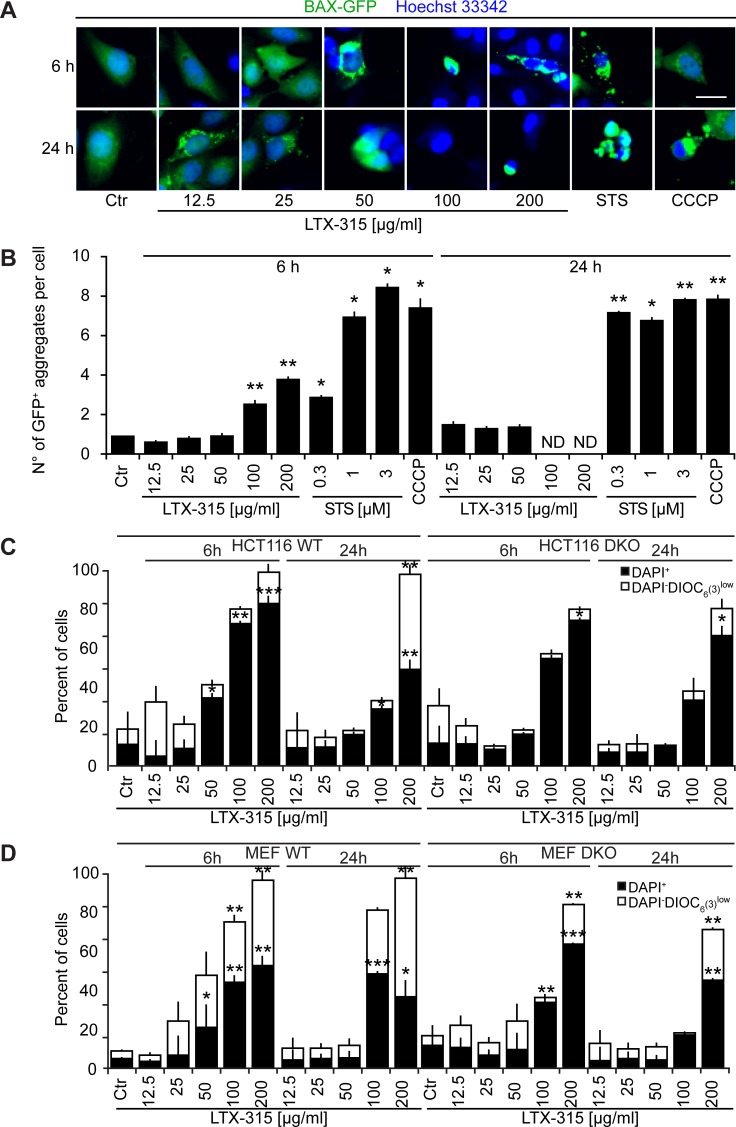
Role of Bcl-2 family protein in cell death induction by LTX-315 **A.**, **B.** Aggregation of BAX-GFP in LTX-315-treated cells. U2OS cells stably expressing a TFAM-GFP fusion protein were cultured for 6 or 24 h with the indicated concentrations of LTX-315, STS (standard dose: 1 μM) or CCCP (standard dose: 10 μM) and then counterstained with Hoechst 33342. Representative pictures of still intact cells are shown in **A.** Quantitative results are shown in **B.** Columns indicate means ± SD of triplicates. Asterisks indicate significant (unpaired Student t test) changes with respect to untreated controls (Ctr). *p < 0.05; **p < 0.01; ***p < 0.001. **C.** Contribution of BAX and BAK to the cytotoxic action of LTX-315. Control cells (left) and double knockout cells (DKO) were cultured for 6 or 24 h with the indicated concentrations of LTX-315, followed by double-staining with DAPI plus DiOC_6_(3) and cytofluorometric detection of dead cells (DAPI^+^) and dying cells (DAPI^+^ DiOC_6_(3)^low^). Results are shown for pairs of WT and DKO HCT116 cells (C) and mouse embryonic fibroblasts (MEF) **D.** Columns indicate means ± SD of triplicates. Asterisks indicate significant (unpaired Welch’s *t* test) changes with respect to untreated controls (Ctr). *p < 0.05; **p < 0.01; ***p < 0.001. Size bar equals 10 μm.

U2OS cells stably transfected with a BAX-GFP fusion protein usually express this fluorescent biosensor in a diffuse location. However, upon induction of apoptosis (with STS) or treatment with the uncoupler CCCP, BAX-GFP aggregates in dense perinuclear areas that likely correspond to mitochondria [26]. LTX-315 was far less efficient in inducing such BAX-GFP aggregates than were STS or CCCP, and only few such effects were found for the intermediate dose of LTX-315 (50 μg/ml) that caused partial cell killing (Figure 6A, 6B). Next, we treated two different double-knockout cell lines (human colon carcinoma HCT116 cells and mouse embryonic fibroblasts, MEF) that lacked both BAX and BAK with LTX-315, comparing the efficiency of cell killing to wild type cells that express BAX and BAK [27, 28]. Although the absence of both BAX and BAK reduced cell killing (measured by staining with the vital dye 4′,6-diamidino-2-phenylindole, DAPI, plus the Δψ_m_-sensitive fluorochrome 3,3′-dihexyloxacarbocyanine iodide, DiOC_6_(3)) to some extent in response to 50-100 μg/ml LTX, cells lacking BAX and BAK still died in response to high doses of LTX (200 μg/ml) (Figure 6C). Altogether, these results suggest that multidomain proteins from the BCL-2 family contribute to LTX-mediated killing.

### Impact of LTX-315 on mitophagy

**Figure 7:**
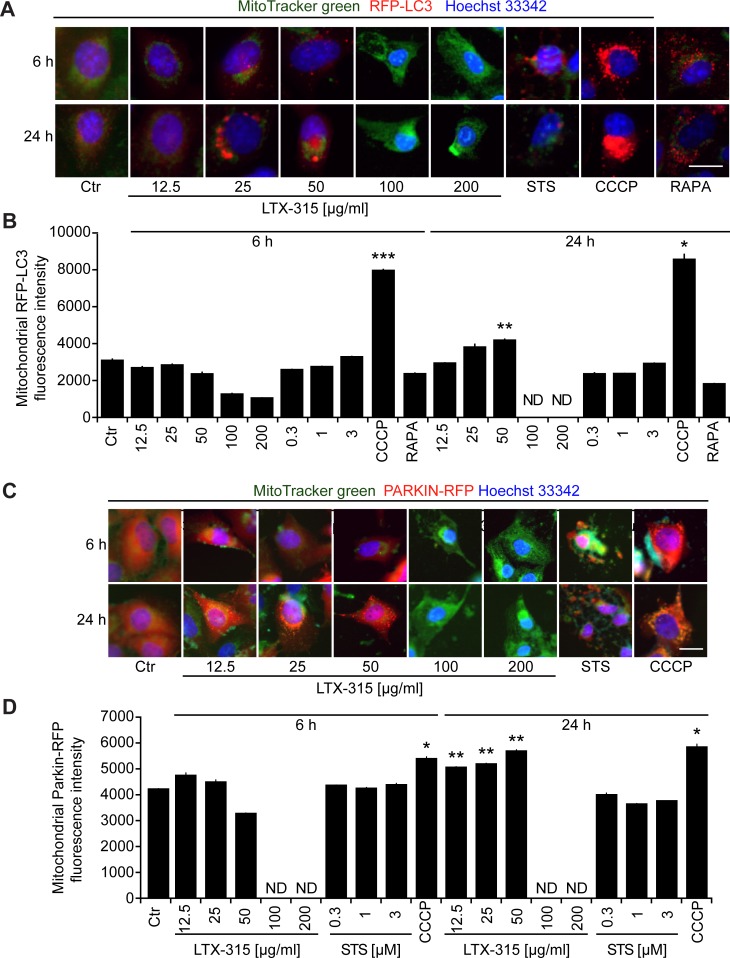
Induction of mitophagy by LTX-315 **A.**, **B.** Sequestration of mitochondria in autophagosomes. U2OS cells stably expressing an RFP-LC3 fusion protein were cultured for 6 or 24 h with the indicated concentrations of LTX-315, STS (standard dose: 1 μM) or CCCP (standard dose: 10 μM) and then counterstained with the mitochondrion-specific dye Mitotracker green, as well as with Hoechst 33342. **C.**, **D.** Translocation of Parkin to mitochondria. U2OS cells stably expressing a mCherry-Parkin fusion protein were cultured as above. Representative pictures of still intact cells are shown in A and C. Quantitative results are shown in B and D. Columns indicate means ± SD of triplicates. Asterisks indicate significant (unpaired Welch’s *t* test) changes with respect to untreated controls (Ctr). *p < 0.05; **p < 0.01; ***p < 0.001. Size bar equals 10 μm.

Mitochondrion-specific autophagy (mitophagy) can lead to the removal of damaged mitochondria, thereby causing resistance to the induction of the intrinsic apoptotic pathway [29-31]. To assess the capacity of LTX-315 to induce mitophagy, we measured the colocalization of an RFP-LC3B fusion protein (which decorates autophagosomes and autophagolysosomes) [32] with mitochondria detected with the ΔΨ_m_-insensitive dye MitoTracker green (MTG). CCCP, which is a strong inducer of mitophagy [30, 33], caused a major co-localization of RFP-LC3B and MTG. In contrast, LTX-315 largely failed to induce such an effect. Only at 24 h, moderately cytotoxic doses (50 μg/ml) of LTX-315 were able to cause a modest mitochondrial localization of RFP-LC3B (Figure 7A, 7B). Mitophagy is accompanied by the translocation of the ubiquitin ligase Parkin from the cytosol to mitochondria [33, 34]. To measure this phenomenon, we generated U2OS cells stably expressing a mCherry-Parkin fusion protein. Short-term treatment (6 h) with CCCP stimulated a significant degree of mCherry-Parkin translocation to mitochondria, which was not found with LTX-315 in the same time range. In contrast, longer treatment (24 h) with low to moderate doses of LTX (12.5 to 50 μg/ml) could induce a minor, though significant degree of mitochondrial relocation of mCherry-Parkin (Figure 7C, 7D). Altogether, these results suggest that LTX-315 can induce a modest degree of autophagy that becomes detectable upon long-term exposure (24 h) to the drug.

### Removal of mitochondria reduced cell killing by LTX-315

**Figure 8:**
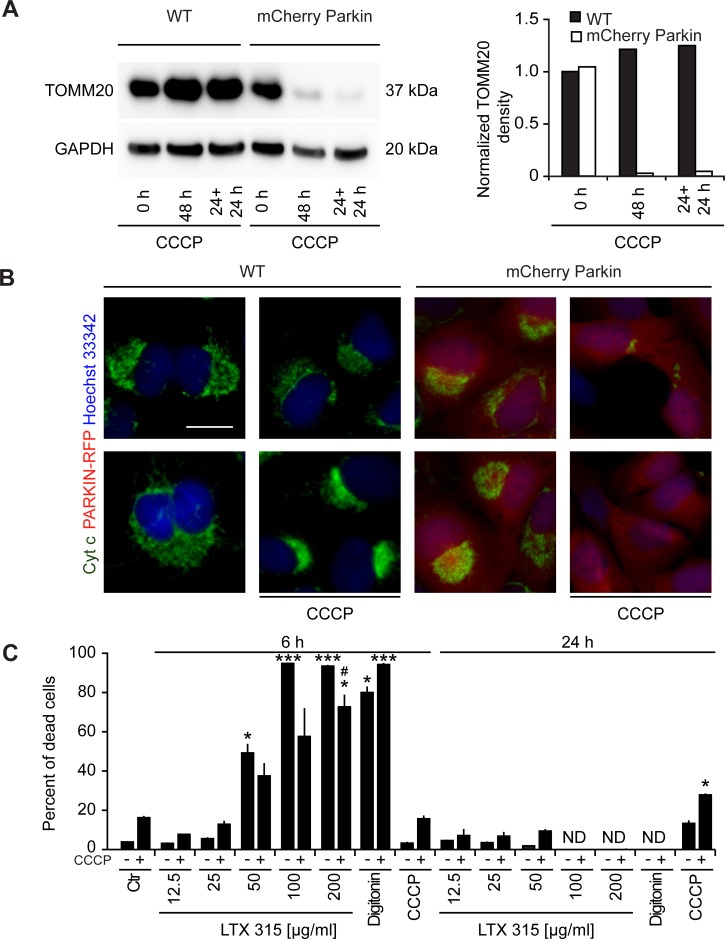
Effect of mitochondrial depletion on LTX-315-induced cell killing **A.**, **B.** Removal of mitochondria by CCCP treatment of U2OS cells stably expressing a mCherry-Parkin fusion protein. WT U2OS cells or cells stably expressing mCherry-Parkin were cultured in the continuous presence of 10 μM CCCP (48 h) or for 24 h, followed by washing and re-culture (24 h + 24 h), followed by immunoblot **A.** or immunofluorescence **B.** detection of TOMM20 or cytochrome **C.**, respectively. **C.** Effect of mitochondrial depletion on the cytotoxic activity of LTX-315. Mitochondria were removed from U2OS cells stably expressing mCherry-Parkin fusion protein by means of the continuous presence of 10 μM CCCP for 48 h. Subsequently the cells were treated with the indicated concentrations of LTX-315, STS or CCCP for additional 6 or 24 h. Following the cells were subjected to microscopical analysis and the percent of cells that depict necrotic phenotypes are shown. Size bar equals 10 μm.

When U2OS cells stably expressing the mCherry-Parkin fusion protein were cultured for a protracted period (48 h) with a high dose of CCCP (10 μM), the cells lost part of the immunoblot-detectable expression of the outer mitochondrial protein TOMM20 as well as that of the intermembrane protein cytochrome *c* (Figure 8A, 8B), suggesting that a significant proportion of the cells are depleted from mitochondria [33]. In a next step, we comparatively assessed the cytotoxic effects of control U2OS cells expressing mCherry-Parkin with mitochondrion-depleted cells (due to pre-treatment with CCCP for 24 h). Importantly, mitochondrion-depleted cells were relatively resistant to the cytotoxic action of LTX-315. In contrast, there was no difference between mitochondrion-positive and mitochondrion-depleted cells with respect to the cytotoxic action of the plasma membrane-permeabilizing agent digitoxin (Figure 8C). These results lend strong support to the idea that LTX-315 kills cancer cells via its mitochondrial effects.

## CONCLUDING REMARKS

The present data indicate that LTX-315 stimulates cell death via its capacity to induce mitochondrial membrane permeabilization. This idea is supported by several levels of correlative evidence, namely (i) the enrichment of the peptide in the mitochondrial fraction of LTX-315-treated cells; (ii) the capacity of LTX-315 to inhibit oxidative phosphorylation; (iii) the ability of LTX-315 to cause mitochondrial fragmentation; (iv) the dissipation of the Δψ_m_ induced by LTX-315; (v) the permeabilization of the outer mitochondrial membrane causing the release of SMAC and cytochrome *c*. All these effects were observed at an LTX-315 dose that was either subtoxic (12.5-25 μg/ml) or partially toxic (50 μg/ml), within cells that otherwise beared a still normal morphology. In addition, two functional manipulations support the idea that LTX-315 kills cells through a mitochondrial mechanisms, namely, (i) the fact that removal of the pro-apoptotic multidomain proteins of the BCL-2 family, BAX and BAK confered protection against LTX-315, and (ii) that depletion of mitochondria by mitophagy reduced LTX-315-mediated cell killing as well. This cytoprotective effect of BAX/BAK or mitochondrial removal was observed at LTX-315 doses of 50-100 μg/ml, which are well below those mediating plasma membrane permeabilization studied in erythrocytes (which lack organelles).

In contrast to the prototype apoptosis inducer staurosporine, LTX-315 is a rather poor inducer of the translocation of BAX-GFP aggregation. Nonetheless we found that LTX-315 was able to induce the formation of BAX-GFP aggregates in at least some cells before they died and that the removal of BAX and of its sessile mitochondrial homologue BAK protects against LTX-315 mediated killing. At present, it remains elusive whether BAK is activated by LTX-315 or whether alternatively, BAX is activated in a transient (‘hit and run’) fashion, as this has been previously suggested [35, 36], meaning that its aggregation becomes not detectable (or perhaps is prevented by the mitochondrion-permeabilizing effect of LTX-315).

While there is little doubt that LTX-315 kills cells secondary to its effects on mitochondria, it is still elusive how the permeabilization of this organelle relays to cell death induction. Indeed, LTX-315 failed to induce the activation of caspase-3 (unpublished data), failed to induce chromatin condensation (as compared to the pro-apoptotic agent staurosporin, Figure 2B, 3A, 4A etc.) and hence did not stimulate an apoptotic death program. In addition, classical experimental interventions designed to block regulated necrosis (such as addition of the RIP kinase inhibitor necrostatin-1 or the cyclophilin D inhibitor cyclosporine A, which inhibits mitochondrial permeability transition) also failed to block LTX-315-induced killing (unpublished data).

High doses of CCCP, which blocked mitochondrial respiration as does LTX-315, yet failed to induce outer mitochondrial membrane permeabilization, had no rapid cytotoxic activity, suggesting that blockade of respiration mediated by LTX-315 is not sufficient to cause cell killing. The comparison of CCCP and LTX-315 reveals interesting particularities of the two distinct mitochondrial toxins. At low doses, CCCP causes mitochondrial uncoupling (and hence an increase in oxygen consumption that can be blocked by respiratory inhibitors), while LTX-315 is unable to do so. CCCP also stimulates massive and rapid mitophagy, which is not the case for LTX-315. Whether the particular mechanism through which LTX-315 permeabilizes (and hence destroys) mitochondrial membranes causes active subversion of the mitophagic pathway remains to be determined.

In conclusion, LTX-315 is an agent that kills cells by distributing to their mitochondria, causing functional and morphological disruption of the organelle, as well as the loss of the barrier functions of mitochondrial membranes. Hence, LTX-315 exemplifies a mitochondrion-targeted oncolytic agent.

## MATERIALS AND METHODS

### Chemicals and cell cultures

Media and supplements for cell culture were obtained from Gibco-Life Technologies (Carlsbad, CA, USA), chemicals from Sigma-Aldrich (St. Louis, MO, USA) with the exception of LTX 315 that was provided by Lytix Biopharma (Tromsø, Norway); CCCP and rapamycin from R&D (Minneapolis, MN, USA), Hoechst 33342, DAPI, DIOC_6_(3), MitoTracker^®^ Green FM, MitoTracker^®^ Orange CMTMRos that came from Life Technologies and plasticware from Greiner Bio-One (Monroe, CA, USA), Ficoll Pacque Plus was from GE Healthcare (Little Chalfont, UK). Primary antibodies (cytochrome C, ab154476 and ab198583; TOMM20 ab78547) were purchased from Abcam (Cambridge; UK) and secondary Alexa Fluor labeled antibodies came from Life Technologies. Human osteosarcoma U2OS WT or syngenic biosensors cells stably expressing Mito-DsRed, SMAC-GFP, PARKIN-mCherry, RFP-LC3, or BAX-GFP were cultured in Glutamax^®^-containing DMEM medium supplemented with 10 % fetal calf serum (FCS), and 10 mM HEPES buffer. Human colorectal carcinoma HCT116 WT and BAX^−/−^,BAK^−/−^ double knockout (DKO) cells were cultured in Glutamax^®^-containing DMEM medium supplemented with 10 % fetal calf serum (FCS), and non essential amino acids. Mouse embryonic fibroblasts (MEF) WT and bax^−/−^,bak^−/−^ double knockout (DKO) cells were cultured in Glutamax^®^-containing RPMI medium supplemented with 10 % fetal calf serum (FCS), and 10 mM HEPES buffer. Cells were grown at 37°C in a humidified incubator under a 5 % CO_2_ atmosphere.

### High-throughput assessment of mitochondrial PARKIN translocation, SMAC release, LC3 lipidation, TFAM release, BAX activation and mitochondrial fission and fusion dynamics

Five x 10^3^ U2OS cells stably expressing PARKIN-RFP; SMAC-GFP; LC3-RFP; TFAM-GFP; BAX-GFP or Mito-RFP were seeded into black 96-well μclear imaging plates (Greiner Bio-One) and allowed to adapt for 24 h. Thereafter the cells were treated with LTX-315 and respective controls and incubated for additional 6 or 24 h before fixation in 3.7 % (w/v) paraformaldehyde in PBS supplemented with 1 μM Hoechst 33342 for 20 min. Upon fixation, PFA was substituted with PBS and a minimum of four view fields per well were acquired by means of an ImageXpress micro XL automated bioimager (Molecular Devices) equipped with a PlanApo 20X/0.75 NA objective (Nikon). For some of the assays cells were stained with 200 nM MitoTracker green or MitoTracker orange (Life Technologies), 1 μg/mL FITC-coupled Phalloidin before imaging.

### Subcellular fractionation

Ten x 10^3^ U2OS cells were seeded in 145 mm cellstar culture dishes (Greiner Bio-One) and allowed to adapt for 24 h. Thereafter the cells were treated with LTX-315 and incubated for additional 6 h before being harvested. Therefore the cells were rinsed with ice cold PBS (pH 7.4) and 3 dishes per condition were collected with a cell scraper in harvesting buffer (PBS, pH 7.4; 1 mM HEPES; 1 mM EDTA). Cells were washed once and the pellet was resuspended in harvesting buffer, subjected to 10 min incubation on ice, and subsequently grinded 100 times on ice using a dounce homogenizer and centrifuged at 700 g for 10 min. The supernatant was recovered and centrifuged at 10000 g for 30 min to obtain the cytosolic fraction (from the supernatant). The pellet was further washed with ice cold PBS and centrifuged 5 min at 450 g. After centrifugation, the pellet was resuspended in 1 mL of cold isolation buffer B (75 mM sucrose; 20 mM Hepes; 225 mM mannitol; 0.5 mM EDTA, pH 7.2), placed on ice and grinded 100 times using a dounce homogenizer and following centrifuged at 750 g for 20 min. The supernatant was re-centrifuged at 10000 g for 10 min to obtain the mitochondrial fraction. The purity of the fractions was analyzed by immunobloting. For detection by mass spectrometry the supernatant of the cytosolic fraction was centrifuged at 10000 g for 1 min at 4°C and 40 μl of supernatant were mixed with 2 μl of formic acid (Sigma Aldrich). The pellet of the mitochondrial fraction was solubilized in 100 μl of water and 40 μl of solution were mixed with 2 μl of formic acid (Sigma Aldrich). The global protein content of each fraction was assessed using the DC protein assay kit from BioRad Laboratories (Hercules, CA, USA) following the manufacture’s recommendations.

### Immunoblotting

Immunoblotting was performed following standard procedures. Briefly, 10 μg of protein were separated on NuPAGE Novex Bis-Tris 4-12 % pre-cast gels (Invitrogen-Life Technologies (Carlsbad, CA, USA)) and transferred to Immobilon polyvinylidene difluoride membranes (Merck-Millipore, Darmstadt; Germany). Unspecific binding was minimized by blocking the membranes for 1 h in 0.05 % Tween 20 (v/v in TBS) supplemented with 5 % w/v bovine serum albumin (Euromedex, Souffelweyersheim, France). Thereafter, membranes were probed with antibodies specific for TOMM20 (Abcam). Primary antibodies were revealed with suitable immunoglobulin G conjugated to horseradish peroxidase (Southern Biotech, Birmingham, AL, USA), followed by chemiluminescence detection with the SuperSignal West Pico reagent in a ImageQuant 4000 (GE Healthcare, Little Chalfont, UK).

### Targeted analysis of intracellular metabolites by UHPLC coupled to a triple quadrupole (QQQ) mass spectrometer

Targeted analysis was performed on a RRLC 1260 system (Agilent Technologies, Waldbronn, Germany) coupled to a Triple Quadrupole 6410 (Agilent Technologies) equipped with an electrospray source operating in positive mode. The capillary voltage was set at 4.5 kV and the gas temperature at 350°C, with a gas flow of 12 l/min. 5 μL of sample were injected on a Column Zorbax Eclipse plus C18 (100 mm x 2.1 mm, particle size 1.8 μm) from Agilent technologies and heated at 40°C. The mobile phase consisted of water 0.2 % acetic acid (A) and acetonitrile (B), with a flow rate set to 0.3 mL/min and initial 90 % phase A and 10 % phase B. Gradient changed as follows: initial conditions were maintained during 1.4 min. Molecules were then eluted using a gradient from 10 % to 95 % phase B over 0.6 min. The column was washed using 95 % mobile phase B for 2.5 minutes and equilibrated using 10 % mobile phase B for 2.75 min. The needle was rinsed with 50 % acetonitrile in water (v/v). The autosampler was kept at 4°C.

MRM transitions were as follows (all in positive mode):

**Table:** 

Compound Name	Precursor Ion (m/z)	Product Ion (m/z)	Fragmentor (V)	Collision Energy (V)
LTX 315 target	360.7	129.1	70	20
LTX 315 qualifier	360.7	443.3	70	15
LTX 315 qualifier	360.7	629.4	70	15
LTX 315 qualifier	360.7	757.4	70	10

### Data processing and statistical analyses

Unless otherwise specified, experiments were performed in triplicate parallel instances and repeated at least once, and data were analyzed with the R software (http://www.r-project.org/). Microscopy images were segmented and analyzed by means of the MetaXpress (Molecular Devices) software and numerical data was further processed with R. Unless otherwise specified, data are presented as means ± SD. Significances have been evaluated using an unpaired Welch’s t test to rectify possible differences in sample variances. Thresholds for the minimum number of events in each analysis necessary to apply further statistics were calculated based on a medium effect size (according to Cohen’s conventional criteria) using the pwr package for R with a targeted value of 0.95. Samples that did not match the requirements were marked ND and were excluded from the analysis.

### Cell death assays

Five x 10^3^ human MEF or HCT116 cells were seeded in 96-well cell culture plates and allowed to adapt for 24 h, then maintained in control conditions or exposed to LTX-315 for 6 or 24 h. For flow cytometry, culture supernatants were transferred to V shaped 96 well plates and cells were detached with 30 μL TrypsinLE™ Express per well, then resuspended in 30 μL of medium and joined with the supernatant in the V-shaped 96 well. The cells were centrifuged 5 min at 200 g and the pellet was resuspended in medium supplemented with 40 nM DiOC_6_(3) and 2 μM DAPI (all from Molecular Probes^®^-Life Technologies, Carlsbad, CA, USA) and incubated for 30 min at 37°C before acquisition. Cytofluorometric acquisition was performed on a CyanADP (Beckman-Coulter).

### Analysis of mitochondrial metabolism

Cellular respiration was measured using the XF-96 analyzer (Seahorse Bioscience, North Billerica, MA, USA). Mitochondrial bioenergetic assays were performed according to manufacurer’s instructions. The XF assay medium (Seahorse Bioscience) was supplemented with 4 mM L-glutamine, 1 mM pyruvate, and 1 g/l D-glucose and pH was adjusted with 1 M NaOH to 7.4 at 37°C. Fifteen x 10^3^ cells were seeded per well and allowed to adapt for 24 h to obtain a monolayer of cells before measurement. After measuring basal respiration the Lytix compounds were injected and a mitochondrial respiration test was performed by sequential additions of 1 μM oligomycin, 0.5 μM carbonyl cyanide-4-(trifluoromethoxy)phenylhydrazone (FCCP) and 1 μM rotenone and antimycin A. Proton leak-induced respiration was calculated as the difference between respiration achieved after oligomycin addition and non-mitochondrial oxygen consumption following rotenone-antimycin A treatment. Maximal respiration induced by FCCP uncoupler was corrected by subtracting the non-mitochondrial respiration values. Subsequently the cells were fixed with 3.7 % of PFA supplemented with 1 μM Hoechst 33342 for 20 min. PFA was substituted with PBS and whole-well imaging was performed by means of an ImageXpress micro XL automated bioimager (Molecular Devices) equipped with a PlanApo 2X/0.1 NA objective (Nikon) followed by enumeration of cells using the MetaXpress software (Molecular devices).

### Mitophagy induction

U2OS cells stably expressing PARKIN-mCherry were treated with 10 μM of CCCP for 48 h for the induction of mitophagy. Thereafter cells were washed and treated with LTX-315 for additional 6 or 24 h. Decreased mitochondrial content was verified by mitochondria-specific anti-TOMM20 or anti-cytochrome *c* immunostaining.

### Erythrolysis assay

Blood was taken from a healthy volunteer. Erythrocytes were purified by Ficoll gradient centrifugation and further diluted (1:1) with PBS. LTX-315 was added and incubated for 4 h at 37°C under a 5 % CO_2_ atmosphere. Supernatants were transferred to microtiter plates and absorbance was measured at 540 nm by means of a SpectraMax i3 multimode plate reader system (Molecular Devices). Images were obtained using a standard HD camera.

### Immunostaining

Five x 10^3^ U2OS cells stably were seeded into black 96-well μclear imaging plates (Greiner Bio-One) and allowed to adapt for 24 h. Thereafter the cells were treated with Lytix-315 and respective controls and incubated for additional 6 or 24 h before fixation in 3.7 % (w/v) paraformaldehyde in PBS supplemented with 1 μM Hoechst 33342 for 20 min. Upon fixation cells were permeabilized with 0,1 % Triton in PBS for 10 min at RT. Unspecific binding was blocked with 2 % BSA in PBS for 10 min at RT followed by primary antibody diluted in 2 % BSA in PBS shaking over night at 4°C. The cells were rinsed twice and stained with AlexaFluor-coupled secondary antibodies for 1 h at RT, rinsed twice and subjected to imaging using an ImageXpress micro XL automated bioimager (Molecular Devices) equipped with a PlanApo 20X/0.75 NA objective (Nikon). For some of the assays cells were additionally stained with 200 nM MitoTracker green or MitoTracker orange (Life Technologies), 1 ug/mL FITC-coupled Phalloidin before imaging.

## SUPPLEMENTARY MATERIAL FIGURES


