# Supplementary material for: The oncolytic peptide LTX-315 kills cancer cells through Bax/Bak-regulated mitochondrial membrane permeabilization
Source: Oncotarget. 2015 Sep 10;6(29):26599–614. doi: 10.18632/oncotarget.5613 (PMC4694939; doi:10.18632/oncotarget.5613)
Supplement: Supplementary file 1 [file oncotarget-06-26599-s001.pdf]

The oncolytic peptide LTX-315 kills cancer cells through Bax/Bak-regulated mitochondrial membrane permeabilization

Supplementary Material

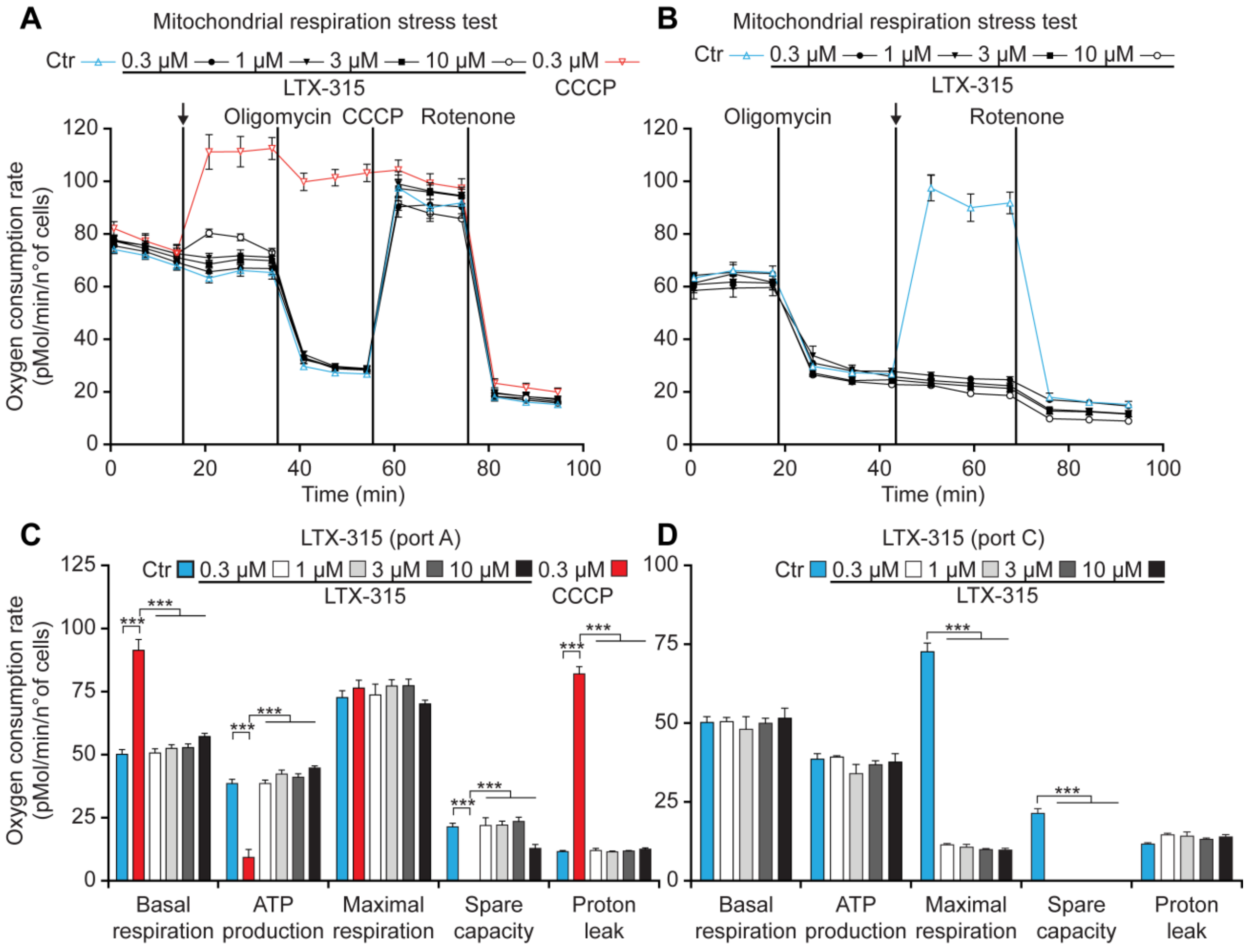

Supplemental figure 1. No stimulation of oxygen consumption by LTX-315. Cells were cultured in specialized XF-96-well plates, and the indicated concentrations of LTX-315 or CCCP were added followed by oligomycin, CCCP and rotenone, as pointed at by the arrows. Oxygen consumption was monitored continuously in a Seahorse XF96 apparatus. Results are means ± SD of hexaplicates.

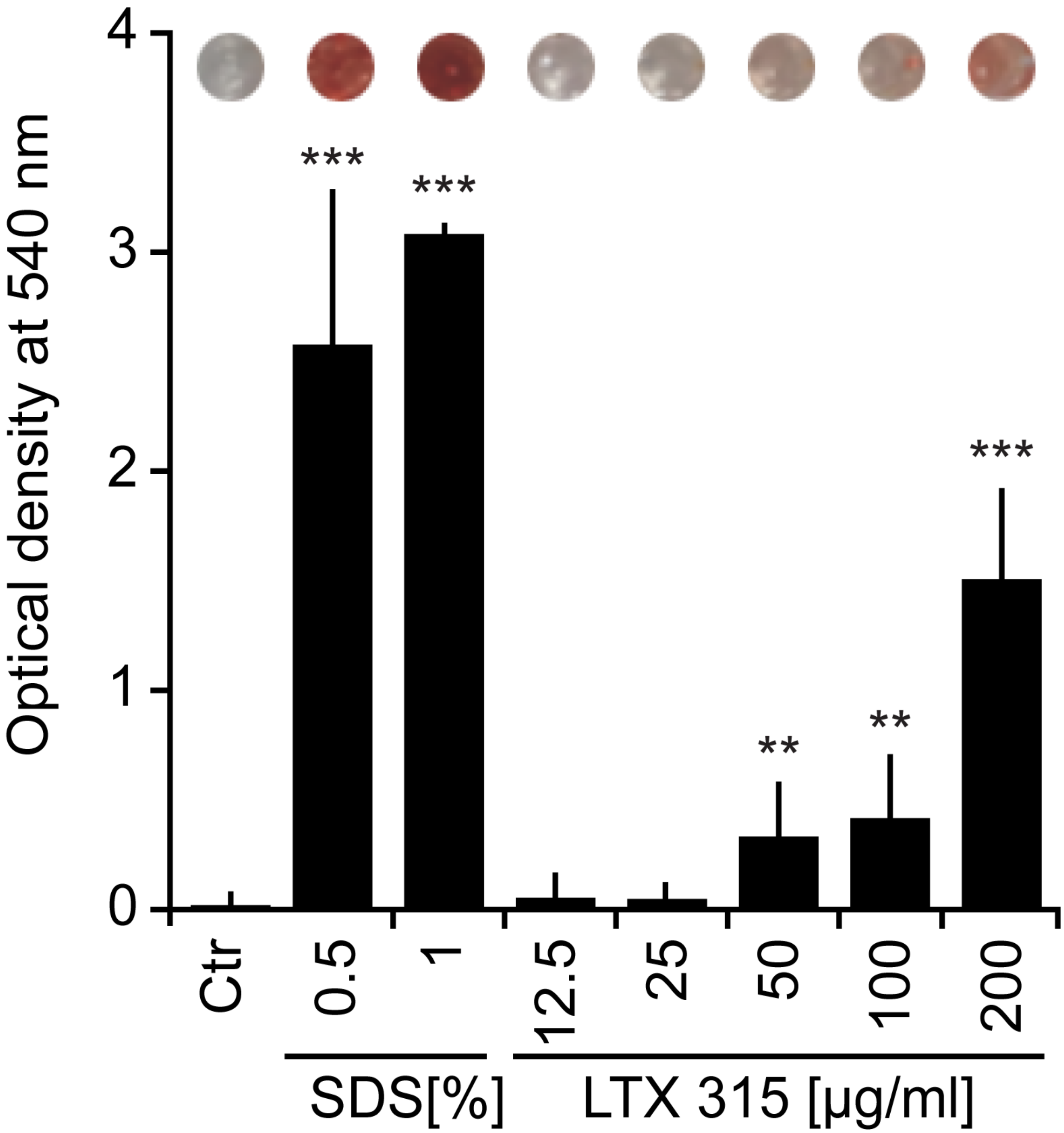

Supplemental figure 2. Partial erythrolysis induced by LTX-315. Erythrocytes were isolated from a healthy donor and were subjected to treatment with the indicated concentrations of LTX-315. Erythrolysis was measured in the supernatants of treated cells via the absorbance detection of released hemoglobin. The inset shows the color of the supernatants of the cells as a sign of hemolysis. Results are means ± SD of quadruplicates. Asterisks indicate significant (unpaired Student t test) changes with respect to untreated controls (Ctr). *$p<0.05$; ** $p<0.01$; ***$p<0.001$.
